# Ligand-Gated Ion Channels as Targets for Treatment and Management of Cancers

**DOI:** 10.3389/fphys.2022.839437

**Published:** 2022-03-08

**Authors:** Rohan Rao, Sanjit Shah, Debanjan Bhattacharya, Donatien Kamdem Toukam, Román Cáceres, Daniel A. Pomeranz Krummel, Soma Sengupta

**Affiliations:** Department of Neurology and Rehabilitation Medicine, University of Cincinnati, Cincinnati, OH, United States

**Keywords:** cancer, genetics, ion channels, membrane protein transport, GABA, ligand receptor

## Abstract

Ligand-gated ion channels are an ionotropic receptor subtype characterized by the binding of an extracellular ligand, followed by the transient passage of ions through a transmembrane pore. Ligand-gated ion channels are commonly subcategorized into three superfamilies: purinoreceptors, glutamate receptors, and Cys-loop receptors. This classification is based on the differing topographical morphology of the receptors, which in turn confers functional differences. Ligand-gated ion channels have a diverse spatial and temporal expression which implicate them in key cellular processes. Given that the transcellular electrochemical gradient is finely tuned in eukaryotic cells, any disruption in this homeostasis can contribute to aberrancies, including altering the activity of pro-tumorigenic molecular pathways, such as the MAPK/ERK, RAS, and mTOR pathways. Ligand-gated ion channels therefore serve as a potential targetable system for cancer therapeutics. In this review, we analyze the role that each of the three ligand-gated ion channel superfamilies has concerning tumor proliferation and as a target for the treatment of cancer symptomatology.

## Introduction

Ionotropic receptors have a variety of triggering mechanisms, including ligand-gated, mechanosensitive, and chemosensitive. The ligand-gated ionotropic channels are highly expressed in a wide variety of tissues and have been successfully targeted to modulate their function since the 1950s (Bianchi and Botzolakis, 2010).

**FIGURE 1:**
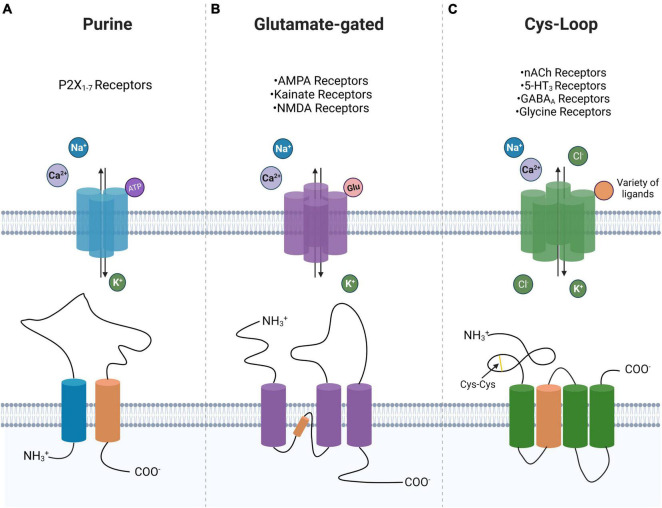
Ligand-gated ion channel superfamily morphology. Shown is a near top-down view displaying preferred ligand and ion selectivity for P2-type ionotropic purinoreceptors **(A)**, glutamate-gated ionotropic receptors **(B)**, and Cys-loop receptors **(C)**. A schematic of the transmembrane domain of each of the three superfamilies is shown with their ligand-interacting domain displayed in orange. Highlighted is the cys-cys loop in the Cys-loop superfamily. Created with BioRender.com.

Ligand-gated ionotropic channel subtypes are commonly classified into one of three superfamilies: ATP-gated or purinoreceptors, glutamate-gated receptors, and Cys-loop receptors (Collingridge et al., 2009). These superfamily classifications are derived from the differing transmembrane domains of each superfamily, which in turn confer functional differences (Table 1). The ATP-gated or purinoreceptors, glutamate-gated receptors, and Cys-loop receptors are trimeric, tetrameric, and pentameric assemblies, respectively (Figure 1).

**TABLE 1:** Ligand-gated ionotropic channel superfamilies.

Receptor subtype(s)	Physiological agonist	Ionic conductance	Select pharmacologic agonists or positive allosteric modulators	Select pharmacologic antagonists or negative allosteric modulators
**Purine**				
P2X_1–7_	ATP	Na^+^. Ca^2+^, K^+^	Beta gamma me-L-ATP	Evan’s blue; PPNDS; Suramin; Minodronic acid; Brilliant blue G (BBG); A438079; AZ10606120
**Glutamate-gated**				
AMPA	L-Glutamate	Na^+^, K^+^, Ca^2+^ (sometimes)	CX1739; L-Quisqualic acid	Perampanel; Telampanel; NBQX; Topiramate
Kainate	L-Glutamate	Na^+^, K^+^	Kainate; (S)-(-)-5-Iodowillardiine	Topiramate; Cyanquixaline (CNQX)
NMDA	L-Glutamate	Na^+^, K^+^, Ca^2+^	Ibotenic acid; Quinolinic acid; Glyx-13	MK-801 (dizocilpine); Memantine
**Cys-loop**				
nACh	Acetylcholine	Na^+^, K^+^, Ca^2+^ (sometimes)	Varenicline; 3-Bromocystine	Pancuronium dibromide; (+)-Tubocurarine chloride; Benzoquinonium dibromide
5-HT_3_	Serotonin	Na^+^, K^+^	Lisuride maleate; Serotonin hydrochloride	Ondasentron; Zacropide hydrochloride; Mirtazapine
GABA_A_	GABA	Cl^–^, HCO_3_^–^	Benzodiazepines	(+)-Bicuculline; Picrotoxin; Furosemide
Glycine	Glycine	Cl^–^	Taurine; uPSEM 817 tartrate	Strychnine; Brucine; Tutin

*P2X, Purinergic 2X; AMPA, α-amino-3-hydroxy-5-methyl-4-isoxazolepropionic acid; NMDA, N-methyl-d-aspartate; nACh, nicotinic acetylcholine; 5-HT_3_, 5-hydroxytryptamine receptor; GABA_A_, gamma-aminobutyric acid Type-A receptor.*

Ligand-gated ionotropic receptors are critical to maintaining a tightly controlled intracellular electrochemical balance in eukaryotic cells. As such, their disruption can lead to inappropriate activation of cellular pathways, including induction of a pro-tumorigenic phenotype. Here, we have taken representative examples from the three ligand-gated ionotropic channel superfamilies to illustrate how they may promote tumor growth and associated tumor symptomatology. Then, we detail the therapeutic potential to focally target the ligand-gated ionotropic channels and thus associated pathways that they contribute to regulating. In discussion of Cys-loop receptors, we highlight how modulating this receptor subtype to perturb an intracellular electrochemical balance in cancer cells can be a successful cancer-specific treatment approach in combination with other therapeutics and/or treatment modalities.

## Purinoreceptors

While adenosine 5’-trisphosphate (ATP) provides intracellular energy to fuel key reactions i.e., phosphorylation, active transport, and motility (Bonora et al., 2012), it also functions as a key signaling molecule (Burnstock, 1972). This signaling is mediated through the action of both metabotropic and ionotropic purinoreceptors. The P1 receptor subclass of purinoreceptors is sensitive to adenosine and displays G-protein coupled receptor (GPCR) activity, while the P2 receptor subclass of purinoreceptors is responsive to adenine and uridine (Campos-Contreras et al., 2020). The P2 subclass is further subclassified into the metabotropic P2Y purinergic receptors (P2YRs), which includes eight mammalian subtypes, and the ionotropic P2X purinergic receptors (P2XRs) with seven mammalian subtypes (Lustig et al., 1993; Brake et al., 1994; North, 2002).

P2X purinergic receptors are widely expressed, including in the brain, peripheral vasculature, lungs, heart, kidney, and immune system with many tissues expressing multiple P2XR subtypes (Huang et al., 2021). P2XRs have been implicated in a variety of intracellular processes ranging from smooth muscle contraction, neurotransmission, macrophage activation, cell proliferation, and death (Burnstock et al., 2010). P2XRs form homo-trimeric (P2X_1_, P2X_2_, P2X_3_, P2X_4_, P2X_5_, P2X_6_, and P2X_7_) or hetero-trimeric (P2X_2/3_ and P2X_1/5_) transmembrane channels, and bind up to three ATP molecules at the interface between adjacent subunits with an EC_50_ on the order of 60 μM (Zhong et al., 1998; Stelmashenko et al., 2012). There is evidence that fewer than three ATP molecules may be sufficient to mediate the opening of the purinergic transmembrane pore (Bean, 1990; Stelmashenko et al., 2012). Upon binding of the ATP ligand, the channel preferentially allows passage of sodium, potassium, and calcium (North, 2016). The P2XR mediated signaling is abrogated by ectonucleotidases, which catalyze ATP breakdown (Novak, 2003).

### Tumor-Related Activity

Purinoreceptors are important to tumor pathogenesis given that ATP is so ubiquitous in the tumor microenvironment (TME) and critical to driving cell proliferation. The concentration of ATP in the TME is approximately 100–500 μM, while 10–100 μM in normal tissues and blood (Hu et al., 2019; De Marchi et al., 2020; Mimoto et al., 2020). The mechanism underlying ATP release in the TME is multifarious as it may be actively secreted, passively released upon cell death, or generated *de novo* extracellularly (Di Virgilio et al., 2018a). Regardless, the dysregulation of extracellular ATP acts in a paracrine or autocrine fashion *via* purinoreceptors to alter the functionality of numerous nearby cell subtypes.

In combination with the changes in extracellular ATP concentration, a variety of P2XRs are upregulated in disparate cancer types, including glioblastoma (GBM), hepatocarcinoma, hepatobiliary carcinoma, breast cancer, multiple myeloma, prostate cancer, and renal cancer (Farrell et al., 2010; Di Virgilio et al., 2018a; Khalid et al., 2018; Asif et al., 2019; Huo and Chen, 2019). The specific tumor type appears to define whether upregulation of P2X may be protective or prohibitive against a tumorigenic phenotype. The mechanism of action of P2XRs in cancer proliferation and growth is multifold. Interestingly, P2XR activation lies on a spectrum as physiological ATP concentrations can promote proliferation and metastasis, whereas excessive ligand binding can induce apoptosis (Virgilio, 2012).

Another effect of P2XRs on cancer pathophysiology is tumor migration and invasiveness. One of the hallmarks of late-stage and metastatic tumors is their ability to spread. This confers a significant disease burden to the tumors as infiltration into non-native tissue can be a common cause of mortality. Therefore, if P2XRs are involved in this process, it is a promising therapeutic avenue to “antagonize” the receptor to diminish tumor motility. In particular, P2XRs have been implicated in tumor epithelial-to-mesenchymal transition (EMT). The EMT confers a morphology to tumor cells that allow for extracellular proliferation and distant metastasis (Thiery, 2002).

Purinergic receptor activation can also secondarily modify tumor proliferation through its activity in the immune system. Firstly, P2XR subtypes are widely expressed in a variety of immune cell subtypes, ranging from macrophages to lymphocytes (He et al., 2017). In human T-cells, ATP release was shown to increase IL-2 expression and p38 MAPK activation (Loomis et al., 2003). IL-2 is a key pro-proliferative cytokine that induces the proliferation of B- and T-lymphocytes. Therefore, increased expression of IL-2 would support an immunogenic response. This conclusion is supported by evidence that “antagonism” of P2X signaling led to T-cell anergy, or functional inactivation (Schenk et al., 2008). The following subsections details the various research on P2XR subclasses and their relationship to cancer pathogenesis.

#### P2X_1_R

One of the key processes necessary for tumor cell survival is a high rate of metabolic activity to outcompete surrounding phenotypically “normal” cells. This is especially highlighted by the Warburg effect in relation to glutamate metabolism (discussed below). Previous data has shown that extracellular ATP acting at the P2X_1_R, along with P2X_7_R and P2YRs, is a trigger for intracellular energy metabolism, enhancing oxidative phosphorylation and subsequent ATP production (Adinolfi et al., 2005; Ledderose et al., 2016; Vultaggio-Poma et al., 2020). The mechanism by which P2XRs are linked to ATP production is an increase in intracellular calcium following purinergic receptor activation which is known to increase mitochondrial metabolic machinery (Jouaville et al., 1999).

#### P2X_2_R

P2X_2_R has not been well researched in regard to cancer pathogenesis but will be discussed further in regard to cancer symptomatology (Kaan et al., 2010).

#### P2X_3_R

While P2X_7_R has been the most extensively researched P2XR subtype in cancer, there have been recent advances implicating P2X_3_R and P2X_4_R as well. High P2X_3_R expression is associated with poor recurrence-free survival in human hepatocellular carcinoma (Maynard et al., 2015). The proposed mechanism is that ATP-mediated activation of P2X_3_R leads to JNK signaling activation which is sufficient to promote cell cycle progression (Maynard et al., 2015).

#### P2X_4_R

Regarding P2X_4_R, the mechanism of action in GBM is activation of P2X_4_R leading to increased brain-derived neurotrophic factor (BDNF)/Trk receptor tyrosine kinase (TrkB) signaling which work in concert to upregulate activating transcription factor 4 (ATF4), a key promoter of cancer cell survival in hypoxic environments (Trang et al., 2009; Singleton and Harris, 2012; Huo and Chen, 2019). Huo and Chen (2019) showed that knockdown of P2X_4_R leads to diminished proliferation and growth in the rat C6 glioma model *via* downregulation of the BDNF/TrkB/ATF4 pathway.

#### P2X_5_R

Epidermal growth factor (EGF)-induced EMT in human breast cancer cells revealed an upregulation of P2X_5_R mRNA, resulting in an increase in ATP-mediated Ca^2+^ signaling (Azimi et al., 2016). However, P2X_5_R was simultaneously downregulated in these same cell lines when exposed to hypoxic environments (Azimi et al., 2016). This further highlights the dynamic and heterogenous nature of cancer cells in which a single receptor subtype can have different mechanisms in different microenvironments.

#### P2X_6_R

In human renal cell carcinoma (RCC) cell lines, P2X_6_R was found to be significantly upregulated compared to HK2 negative control cells (Gong et al., 2019). Upon ATP activation, P2X_6_R-mediated Ca^2+^ entry induced activation of p-ERK1/2-MMP9 axis to promote RCC metastasis and invasion (Gong et al., 2019). The activation of calcium-mediated ERK1/2 pathway is a unifying factor with the P2X_5_R and P2X_7_R putative mechanisms in cancer proliferation. This necessitates further research on a combinatorial therapeutic approach blocking multiple channels’ activity by targeting the ultimate downstream targets. Moreover, further research needs to be performed on the P2X_6_R subclass on whether its expression is altered in hypoxic environments like the P2X_5_R.

#### P2X_7_R

Within the P2XR subclass, the P2X_7_R subtype is perhaps the most well studied in regard to cancer pathogenesis (Di Virgilio et al., 2021; Hreich et al., 2021; Matyśniak et al., 2021). Regarding proliferation, it has been shown that the P2X_7_R subtype initiates an intracellular phosphorylation cascade resulting in the activation of the PI3K/Akt and ERK1/2 pathways, key regulators of cancer survival (Amoroso et al., 2015; Salahuddin et al., 2021). To prove this point, Qiu et al. (2014) demonstrated in human prostatic carcinoma cell lines that knockdown of P2X_7_R led to diminished PI3K/Akt and ERK1/2 pathway activation in response to exogenous ATP; this, in turn, decreased tumor proliferation and growth. The PI3K/Akt pathway has a reciprocal effect on P2X_7_Rs by increasing the transcription of the *P2rx7* gene through the action of specificity protein 1 (Sp1) (Gómez-Villafuertes et al., 2015). This potentially serves as a positive feedback loop in cancer proliferation in which there is a continuous loop of increased extracellular ATP concentration leading to increase purinergic receptor activation followed by PI3K/Akt pathway activation. The activation of PI3K/Akt also suggests that P2X_7_R may help maintain stem cell-like populations in tumors (Rabelo et al., 2021). It has also been shown in human embryonic kidney cells that P2X_7_R-expressing tumors were characterized by an increased expression of the transcription factor NFATc1 which conferred increased proliferative capability (Adinolfi et al., 2012). The theme that persists even in purinergic signaling is that different receptor subtypes can have differing effects on different tumors.

P2X_7_R knockdown has been shown to reduce the expression of Snail, E-cadherin, Claudin-1, interleukin (IL)-8, and MMP-3 which are all EMT-related genes (Qiu et al., 2014). Another study showed that direct activation of P2X_7_R led to an upregulation of all forms of mature cysteine cathepsins which are proteases dedicated to the breakdown of the extracellular matrix (Jelassi et al., 2011). This in turn conferred increased invasiveness in human-derived breast cancer cells. Interestingly, the pro-tumorigenic activity of P2X_7_R in tamoxifen-resistant breast cancer cells was found to be linked with migration and metastasis independent of matrix metalloproteinase (MMP) signaling and EMT factors. The P2X_7_R-mediated migration in tamoxifen-resistant breast and melanoma cancer cells was found to be linked with miRNA-containing small extracellular vesicles (sEV) (Park et al., 2019; Pegoraro et al., 2021). Again, it is highly likely that the differential activation of P2XR subtypes leads to the activation of different intracellular pathways which all converge to increase tumor motility and proliferation.

**FIGURE 2:**
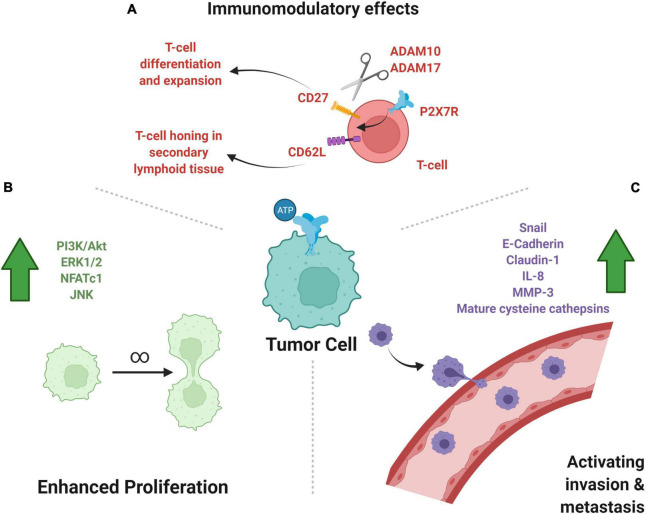
P2-type ionotropic purinoreceptors in cancer growth, metastasis, and immunomodulation. **(A)** Shown are the anti-tumor, pro-immunomodulatory effects of P2XR activity on immune cells. The effect on T-cells of P2X_7_R activity leads to cleavage of CD27 and CD62L, which help in T-cell clonal expansion and honing to target tissue, respectively. **(B)** Subcellular pathways upregulated by purinergic signaling may lead to increased cell division and tumor growth, as illustrated. **(C)** P2X signaling upregulates molecules that favor metastasis, a key prognostic factor into the severity of tumor disease burden. Created with BioRender.com.

Another interesting role of purinoreceptors in immunomodulation is the activation of metalloproteases, ADAM10 and ADAM17, by P2X_7_Rs which leads to the shedding of CD62L, CD27, and IL-6R (Gu et al., 1998; Di Virgilio et al., 2018b). CD62L, also known as L-selectin, is critical for circulating lymphocytes to recognize endothelial cells in a target tissue, allowing for lymphocytes to enter secondary lymphoid organs (Yang et al., 2011). CD27 is a member of the TNF receptor superfamily and upon binding of its ligand, CD70, is thought to play a key role in B-cell activation and the differentiation and clonal expansion of T-cells (Hendriks et al., 2000; Grassi and De Ponte Conti, 2021). The combination of these cleavages implicates purinergic receptor activation in an increased immune response to cancer cells (Cai et al., 2021). The combination of pro-proliferative and migratory effects of purinergic signaling and the pro-immunomodulatory response makes purinergic signaling a particularly interesting drug target (Figure 2).

### Therapeutic Potential

As with our discussion of other ligand-gated ionotropic receptors (below), we turn to recent developments in P2XR “antagonism” for tumor therapy. While purinergic signaling is a more recent target for chemotherapeutics, the targeting of purine/purinergic incorporation into rapidly dividing cancer cells has been a highly utilized cancer therapeutic mechanism. For example, the 5-Fluorouracil (5-FU) prodrug, capecitabine, both incorporate incorrect fluoronucleotides into RNA and DNA while simultaneously inhibiting the action of thymidylate synthase (Longley et al., 2003). By this mechanism of action, rapidly dividing cancer cells are preferentially killed. Returning to purinergic signaling, much of the literature surrounding P2XR “antagonism” concerns abrogation of the P2X_7_R subtype. In a rat model of C6 glioma, pharmacological inhibition of P2X_7_R by brilliant blue G (BBG) inhibited tumor growth (Ryu et al., 2011). Mohammed et al. showed that the use of the P2X_7_R antagonists, A438079 and AZ10606120, reduced caspase-3, caspase-1, p21, and Cdc25c in a mouse model of pancreatic cancer which resulted in decreased cancer proliferation (Mohammed et al., 2017; Zhang et al., 2021). Similarly, B16 melanoma cells and B16 melanoma-bearing mice when treated with oxidized ATP (purinergic receptor inhibitor) displayed decreased tumor cell proliferation (Hattori et al., 2012). However, one must also weigh the anti-immune effects of P2X antagonism when considering these anti-tumorigenic effects.

### Symptom Management

While having anti-tumorigenic potential, purinergic therapeutics also may be used in symptom management. Different types of pain are associated with P2XR activation. For example, the P2X_3_R subtype expressed on sensory neurons has been associated with the transmission of neuropathic pain (Bele and Fabbretti, 2015). Similarly, inhibition of the P2X_4_R subtype in spinal cord microglia through intrathecal injection of bone marrow stromal cells attenuated neuropathic pain in a murine model (Ulmann et al., 2008; Teng et al., 2019). The mechanism of this nociception is thought to be activation of P2X_4_R on microglia by afferent neurons leading to MAPK signaling, which in turn leads to the synthesis and excretion of brain-derived neurotrophic factor (BDNF) (Ulmann et al., 2008; Trang et al., 2009).

A specific subtype of cancer-related pain is cancer-induced bone pain (CIBP) which represents a significant burden on the quality of life. CIBP is the product of metastasis of non-bone tumors to the bone resulting in debilitating pain. Interestingly, antagonism of the P2X_2/3_R by minodronic acid, a third-generation bisphosphonate, resulted in an analgesic effect in a murine model of CIBP as measured by nociceptive behaviors (Kakimoto et al., 2008).

## Glutamate Receptors

Like other ligand-gated ionotropic channel superfamilies, the glutamate family of receptors has metabotropic, metabotropic glutamate receptor (mGluR), and ionotropic, ionotropic glutamate receptor (iGluR), subfamilies. The three major receptors in the iGluR subfamily are the *N*-methyl-*D*-aspartate (NMDA), alpha-amino-3-hydroxyl-5-methyl-4-isoxazole-propionate (AMPA), and kainite receptors, so named for their endogenous agonists (Table 1; Purves et al., 2001). Ionotropic glutamatergic signaling has been well studied for its role in the CNS as the source of rapid depolarizing signaling originating from excitatory neurons. Upon binding of ligand, all three channels allow for the selective conductance of K^+^ and Na^+^ ions—with small amounts of Ca^2+^ in the case of the NMDA channel. Interestingly, the NMDA channel’s pore is blocked by a Mg^2+^ ion at hyperpolarized states but is removed when depolarized. Moreover, NMDA requires the co-activator glycine for its full conductance to be achieved. With the influx of Ca^2+^, NMDA channels can activate other intracellular signaling cascades, implicating NMDA channels in a variety of longer-term synaptic processes such as learning, memory, and neuroplasticity (Blanke and VanDongen, 2009). The slower activation of NMDA receptors has led to the conclusion that they are not involved in the generation of action potentials (Yi et al., 2020). On the other hand, due to the lack of a co-activator or pore blocker, the AMPA and kainate receptors have been shown to produce very fast excitatory post-synaptic currents (EPSCs). The ligand-binding domains are highly conserved across iGluR subfamilies and are classically referred to as S1 and S2 (Stern-Bach et al., 1994).

The structure of iGluRs consists of a tetrameric transmembrane domain surrounding a central pore (Figure 1). The NMDARs are constructed from several possible subunits including GluN1, GluN2A, GluN2B, GluN2C, GluN2D, GluN3A, and GluN3B (Kew and Kemp, 2005; Mayer, 2005; Bettler et al., 2019). The NMDAR consists of two requisite GluN1 subunits with any remaining subtype comprising the final two subunits of the tetramer (Paoletti et al., 2013). The GluN1 subunit determines calcium conductance, whereas the GluN2 and GluN3 subunits determine the pharmacological and electrophysiological properties of the channel. The AMPAR arises from a combination of GluA1, GluA2, GluA3, and GluA4 subunits. Kainate receptors are assembled from GluK1, GluK2, GluK3, GluK4, and GluK5 subunits that can form either a homotetramer of GluK1-3 subunits or a heterotetramer of GluK1-5 subunits (Stepulak et al., 2014; Bettler et al., 2019). Again, alternative splicing allows for another layer of heterogeneity upon this pre-existing channel subunit complexity.

Glutamate receptors are physiologically expressed in a more limited range of tissues, compared to the other superfamilies discussed in this review. Glutamate receptor tissue expression is observed primarily in the brain, spinal cord, retina, and peripheral nervous system (Traynelis et al., 2010). On the other hand, iGluRs are expressed in disparate tumor subtypes, being upregulated in CNS tumors, colorectal, hepatocellular, gastric, thyroid, larynx, melanoma, osteosarcoma, and blood neoplasms (Ganor et al., 2009; Stepulak et al., 2009; Li et al., 2012; Stepulak et al., 2014; Gong et al., 2017). Given the numerous linkages with various iGluRs in cancer, this represents a potentially highly targetable system to inhibit cancer growth (Rzeski et al., 2001). The endogenous ligand, glutamate, is synthesized by the deamination of glutamine (Schousboe et al., 2014; Yi et al., 2020). Glutamate is a key intermediary in a variety of anabolic reactions with the possibility of creating gamma-aminobutyric acid (GABA), suggesting an interplay between the two signaling pathways in cancer (see below). The additional deamination of glutamate can be used in the synthesis of purines and pyrimidines and the carbon skeleton can be incorporated into ATP. Similarly, the carbon skeleton of glutamate can be used to make other amino acids such as ornithine, arginine, and proline. Of particular relevance to this review is that glutamate is also a precursor to glutathione which is a reaction oxygen species (ROS) scavenger. As will be discussed further, this allows for cancer cells to survive oxidatively stressful environments. The dual-activity as both a key anabolic intermediate and a signaling molecule gives glutamate a critical role in tumor pathogenesis.

### Tumor-Related Activity

Particularly unique to the discussion of the glutamatergic superfamily is the fact that the ligand, glutamate, is not only implicated as an extracellular messenger but also as a key intracellular modulator of tumor proliferation. Otto Warburg noticed that cancer cells take up glucose at almost a tenfold rate compared to normal cells and shunt glucose through the anaerobic pathway to produce lactate (e.g., the Warburg effect) (Koppenol et al., 2011; Liberti and Locasale, 2016). As glucose is frequently shunted to glutamate, it is natural that increased glutamate levels in various cancers correlate to poorer prognoses. For example, in prostate cancer, glutamate concentrations directly correlated with advanced Gleason scores, a clinical prostate staging system (Koochekpour et al., 2012). Researchers are also analyzing whether glutamate levels can be used as a diagnostic approach in breast cancers due to a similar logic as in pancreatic cancers (Budczies et al., 2015). The tumor microenvironment generates high concentrations of ROS. Basal levels of ROS lead to cancer proliferation, whereas high levels of ROS induce a cytotoxic response (LeBoeuf et al., 2020). Cancer cells co-opt the elevated levels of glutamate to produce glutathione which can act as a “sponge” of ROS to maintain the optimal range of ROS concentration, allowing for cancer growth (Hayes et al., 2020).

#### *N*-Methyl-*D*-Aspartate

Returning to the mechanism of glutamate binding to its receptor, the variety of glutamatergic signaling in cancer impacts multiple intracellular pathways. One well-studied association is the activation of NMDA receptors leading to activation of the mTOR pathway, which is classically associated with cell proliferation; the mechanism by which glutamate binding to NMDA activates mTOR is still debated with the two prevailing hypotheses being a Ca^2+^ mediated activation of p-ERK or *via* inhibition of membrane cationic amino acid transporters (Huang et al., 2007; Tang et al., 2015). Interestingly, the NMDA-mediated activation of the p-ERK pathway gives a potential connection to the P2XRs which operate on the same pathway. NMDA receptors are important regulators of pancreatic cancers and pharmacological blockade of NMDA receptors, especially the GluN2B sub-unit, was found to have significant growth inhibitory effects on human pancreatic tumor cells in the orthotopic xenograft mice model (North et al., 2017). Furthermore, NMDA receptors have been linked to activation of ERK signaling and cAMP-response element-binding protein (CREB) in human non-small cell lung cancer (NSCLC) cells. The non-competitive NMDA receptor antagonist MK-801 (dizocilpine) was reported to reduce phosphorylated ERK1/2 levels in a concentration-dependent manner (Stepulak et al., 2005). Further, treatment of NSCLC cells with the NMDA receptor antagonist MK-801 was shown to decrease phosphorylation of CREB, which is involved in regulating transcription of various cell cycle genes including p21 (Stepulak et al., 2005). Functional NMDA receptors were also found to be expressed in patient-derived human small cell lung cancer (SCLC) cell lines NCI-H345, DMS-53, NCI-H146, and NCI-H82 as well as in SCLC tissue sections (North et al., 2010). *In vivo*, MK-801 was shown to inhibit the growth of tumor xenografts of H345 SCLC cells (North et al., 2010). Finally, research on the repositioning of the Alzheimer’s drug memantine (an NMDAR antagonist) showed inhibition of cell cycle progression of prostate cancer cells (Albayrak et al., 2018). The mechanism of action was through upregulation of the Bax-dependent apoptotic pathways.

#### Alpha-Amino-3-Hydroxy-5-Methyl-4-Isoxazolepropionate

Regarding AMPAR, Ishiuchi et al. (2007) showed that glutamate released by glioblastoma cells acts in a paracrine or autocrine fashion to activate AMPAR which in turn led to the influx of Ca^2+^ allowing for the phosphorylation of Akt at Ser-473. The activation of Akt supports glioblastoma proliferation and growth and has been well studied in relation to the tumor suppressor PTEN (Choe et al., 2003). Not only is glutamate released by glioma cells, but there exists neuron-to-glioma synaptic transmission in which neuron-mediated glutamate release and subsequent binding to AMPAR on glioma cells led to increased proliferation *in vitro* (Venkatesh et al., 2019). In a mouse model of pediatric glioma, AMPAR blockade through perampanel, a non-competitive antagonist of AMPAR, led to a 50% decrease in glioma growth in perampanel-treated mice compared to vehicle control (Venkatesh et al., 2019). In pancreatic cancer, activation of AMPAR led to increased MAPK signaling and K-Ras signaling which promoted invasion and migration (Herner et al., 2011). RNAi suppression of glutamatergic signaling suppressed the observed AMPAR effects on pancreatic cancer cell migration. Similarly, antagonism of AMPA signaling in human lung adenocarcinoma cells led to a restoration of the tumor suppressor p21 and p53 with a concomitant halt in lung cancer growth (Stepulak et al., 2007).

#### Kainate

Lastly, the kainate receptor also has been linked to the breast cancer epithelial-to-mesenchymal transition (EMT). Firstly, Xiao et al. (2019) found these kainate receptors to be significantly upregulated in human-derived breast cancer cells. The mechanism by which kainate receptors were shown to increase the EMT transition was through the upregulation of SPDEF/CDH1 signaling (Xiao et al., 2019). As mentioned previously, the EMT transition is critical to tumor proliferation and migration.

### Symptom Management

**FIGURE 3:**
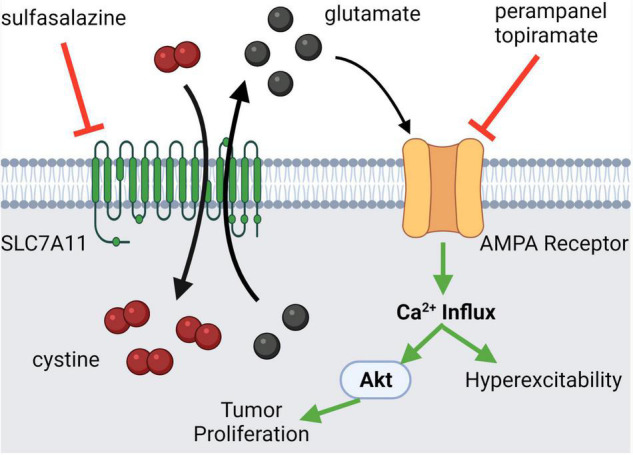
Glutamatergic signaling in central nervous system (CNS) tumor-induced seizures. Many CNS tumors present with seizures due to mass effect or increased extracellular glutamate release mediated by the SLC7A11 channel, a cystine-glutamate exchanger. The excess glutamate in the extracellular fluid binds to AMPA receptors autologously, leading to Ca^2+^ influx which may activate pro-tumorigenic pathways. Alternatively, the excess glutamate may act in a paracrine fashion, leading to nearby neuronal hyperexcitability, which manifests clinically as a seizure. Antagonists of both the SLC7A11 channel and the AMPAR can modulate this pathological state. Created with BioRender.com.

Seizures are one of the most common presenting features of CNS tumors. It is thought that the release of glutamate from the tumor microenvironment (TME) *via* a cystine-glutamate exchanger (SLC7A11, xCT) contributes to this clinical presentation (Sørensen et al., 2018). It has been shown that high levels of glutamate induce seizures through its excitatory effect (Buckingham et al., 2011). Perampanel, a non-competitive AMPAR antagonist, has already been established to treat patients with seizures (Lange et al., 2021). Therefore, it can be easily translated to cancer-induced seizures with the potential added benefit of inhibiting tumor progression through the aforementioned mechanisms. Aside from directly antagonizing the iGluRs, SLC7A11/xCT can be antagonized through sulfasalazine (cystine-glutamate exchanger antagonist) which reduces extracellular glutamate release, thereby decreasing seizure incidence (Figure 3; Verbruggen et al., 2021). An additional benefit of this therapeutic modality is that there is potential for a synergistic anti-tumor effect, treating the cause and symptoms simultaneously. Highlighting this point, SLC7A11 antagonism caused synthetic lethality in *KRAS-*mutant lung adenocarcinoma (Hu et al., 2020).

Glutamatergic signaling has also been shown to play a critical role in the peripheral sensitization and mechanical hypersensitivity associated with CIBP. Zhu et al. (2020) showed that glutamate injection-induced neuronal excitation in dorsal root ganglia (DRG) is similar to that shown to be induced by CIBP. In a breast cancer model of CIBP, sulfasalazine reduced nociceptive perception and delayed onset of behavioral pain signs (Ungard et al., 2014; Ellingson and Vanderah, 2020; Zhu et al., 2020). Furthermore, in a rat model of CIBP, direct inhibition of NMDAR in the spinal cord led to nociceptive relief (Dai et al., 2017). The proposed mechanism of action was through reduced phosphorylation of PKCγ and ERK1/2 in the spinal cord (Dai et al., 2017).

## Cys-Loop Receptors

Cys-loop receptors are so named for the presence of a disulfide bridge in a loop of its extracellular domain (Figure 1; Miller and Smart, 2010). Canonical Cys-loop receptors include nicotinic acetylcholine (nACh), 5-hydroxytryptamine receptor (5-HT_3_/serotonin), the gamma-aminobutyric acid Type-A receptor (GABA_A_R), and glycine receptor. The ionic conductance of the Cys-loop receptors varies between the subtypes. For example, nicotinic acetylcholine and serotonin (5-HT_3_) receptors are cationic-selective channels that selectively depolarize the cell. On the other hand, GABA_A_Rs and glycine channels are anionic-selective which serve to hyperpolarize developmentally mature cells.

All Cys-loop receptors share a similar superstructure: an extracellular domain for ligand binding; a transmembrane domain for ion passage; and an intracellular domain for the potential regulation of channel conductance (Thompson et al., 2010). The transmembrane portion of the receptor consists of a pentameric topology/structure with a central pore for ion passage upon binding of the ligand (Figure 1). We focus on the GABA_A_R due to its proposed role in cell proliferation which highlights it as a potential pro-cancerous ligand-gated ionotropic channel (Takehara et al., 2007; Young and Bordey, 2009; Bhattacharya et al., 2021).

The endogenous GABA_A_R ligand, GABA, is a product of the decarboxylation of glutamic acid catalyzed by glutamic acid decarboxylase (GAD65, GAD67) (Olsen and DeLorey, 1999). Transcript levels of GAD are thus frequently used as a proxy for the identification of GABAergic neurons. The action of GABA is abrogated by its metabolism by GABA transaminase (Olsen and DeLorey, 1999; Young and Bordey, 2009). GABA is thought to regulate cell dynamics through at least two receptor subtypes—the GABA_A_R and the GABA_B_R, which is a metabotropic G-protein coupled receptor (GPCR).

**FIGURE 4:**
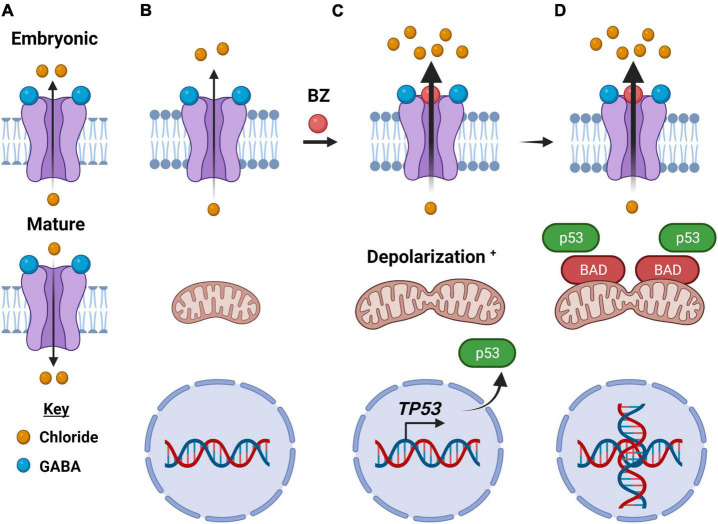
How type-A GABA receptors may function as an electrochemical vulnerability to cancer cells. **(A)** In an embryonic cell, the GABA_A_ receptor upon binding of GABA moves chloride anions out of the cell and is depolarizing (top), while in a mature neuron, GABA_A_ receptors move chloride anions into the cell upon binding of GABA and is hyperpolarizing (bottom). **(B)** In cancer cells, the direction of ion flow is out of the cell, like that of the embryonic receptor. **(C)** Anion flow is enhanced upon binding of a benzodiazepine, a positive allosteric modulator of the GABA_A_ receptor. Within minutes of benzodiazepine binding to the GABA_A_ receptor, the significant efflux of chloride anions depolarizes the mitochondrial transmembrane as well causes their fission. In addition, there is enhanced expression of the oncogene TP53. **(D)** The intrinsic (mitochondrial) apoptotic pathway is activated, including an enhanced expression and localization of the BCL2 Associated Agonist of Cell Death (BAD) protein. There are also significant morphological changes in the cancer cells, including DNA duplication, as illustrated. Created with BioRender.com.

Even within the GABA_A_R subtype there exists significant compositional heterogeneity as 19 receptor subtype genes have been identified in humans, coding for six α, three β, three γ, three ρ, one δ, one ε, one π, and one θ-subunit (Sigel and Steinmann, 2012; Ghit et al., 2021). The most common adult human assembly is of a hetero-pentamer consisting of two α, two β, and one γ-subunit encoded by *GABR* genes *GABRA*, *GABRB*, and *GABRG*, respectively. The ligand (GABA) binding site is at the interface between α- and β-subunits (Figure 4; Olsen, 2018; Bhattacharya et al., 2021). Normally, a GABA_A_R allows for the passage of chloride anions intracellularly through the transmembrane pentameric pore following the binding of GABA. This in turn leads to a hyperpolarization of the mitochondrial transmembrane, which is characteristically associated with neuro-suppressive effects in the central nervous system (CNS). However, critical to this discussion is the “GABA switch.” During the post-natal period, neurons change the intracellular concentration of chloride anions [Cl^–^] due to the expression of certain channels. In particular, the Na^+^-K^+^-2Cl^–^ (NKCC1, SLC12A2) transporter is highly expressed in immature neurons and is responsible for the high intracellular [Cl^–^]. The delayed expression of the K^+^-Cl^–^ cotransporter (KCC2) is thought to be responsible for the reduction in intracellular [Cl^–^] (Kandler and Friauf, 1995). As a product of an increased extracellular to the intracellular ratio of chloride anion, GABA_A_Rs act to hyperpolarize rather than depolarize the cell following the GABA switch (Ganguly et al., 2001). Interestingly, GABA_A_R in cancer cells behaves similarly to GABA_A_Rs in immature, developing neurons as assessed in part by patch-clamp electrophysiology (Labrakakis et al., 1998; Sengupta et al., 2014; Kallay et al., 2019). Given that cell depolarization is commonly associated with proliferative pathways linked to malignant transformation, this detail is key to the hypothesized role of GABA_A_Rs in cancer.

Gamma-aminobutyric acid type-A receptor is not functionally distinct to the CNS. GABA_A_R subunits are expressed throughout the body, including in the lungs, pancreas, kidney, intestine, prostate, and skin (Napoleone et al., 1990; Gilon et al., 1991; Borboni et al., 1994; Jin et al., 2008; Takano et al., 2014) where they have been shown to contribute to a diverse range of functions, including modulating proliferation, regulating secretory function, and vascular control (Ong and Kerr, 1990; Erlitzki et al., 2000; Watanabe et al., 2002; Bansal et al., 2011).

### Tumor-Related Activity

GABA_A_R subunits expression is enhanced in both CNS and systemic cancers, including pediatric and adult brain tumors (Cho et al., 2011) and gastric, pancreatic, ovarian, and breast cancers (Hujber et al., 2018; Jiang et al., 2020). In addition, GABA_A_Rs are not only significantly upregulated in medulloblastoma, a pediatric brain cancer, and melanoma but also are a functional GABA-responsive receptor (Sengupta et al., 2014; Pomeranz Krummel et al., 2021). The putative pathway of GABA in the proliferation of these cancers has yet to be fully elucidated, but one prevailing theory is that the lack of a GABA switch with subsequent retention of a depolarizing phenotype in many cancers allows for pro-tumorigenic pathways to be upregulated. In support of this theory is research done on pancreatic cancer cells which showed that enhancing GABA_A_R activity with an agonist-induced a Ca^2+^ influx, resulting in subsequent MAPK/ERK pathway activation (Takehara et al., 2007). This MAPK/ERK activation was reduced by the administration of a calcium chelator, further implicating calcium in this pathway. Aside from inducing proliferation, GABAergic signaling and metabolism may aid tumor metastases to survive in the brain microenvironment. Neman et al. (2014) showed that breast-to-brain metastatic cells displayed increased GABA metabolism and a strong association between HER2 and reelin, a protein expressed by GABAergic neurons, implicating this phenotype with control over cell motility and cytoskeletal shape. These studies imply that a hyperactivation of GABA_A_R may be partially responsible for a tumorigenic phenotype.

Conversely, other research has shown that GABA agonism may inhibit cancer proliferation and growth which complicates the therapeutic landscape given the discrepancy with the aforementioned literature. GABA signaling has been implicated in the maintenance of embryonic (ESC) and neural stem cells (NSC) in the stem cell niche, preventing proliferation (Wang et al., 2008). Paradoxically, this mechanism is found to be a hyperpolarizing effect of GABA_A_R despite occurring in stem cells, which presumably have not undergone a GABA-switch (Wang et al., 2008). GABA_A_R is thought to activate S-phase checkpoint regulatory kinases such as PIKK which in turn phosphorylate the histone H2AX, correlated with decreased proliferation in ESC and NSC cells (Andäng et al., 2008). This mechanism may provide an explanation as to why in human hepatocellular carcinoma, prostate cancer, and some brain tumors the downregulation of GABA_A_R allows for increased tumor proliferation (Young and Bordey, 2009).

### Therapeutic Potential

Given GABA’s documented role in proliferation and stem cell maintenance, is the GABAergic system a possible target for anti-tumor activity? GABA_A_Rs are already a highly targeted neurotransmitter receptor subtype for the treatment of anxiety, insomnia, and epilepsy. Although benzodiazepines have received “bad press” as of late, due to their addictive potential (Oransky, 2005). An epidemiological study conducted by Kleinerman et al. revealed that patients on diazepam fared better, e.g., exhibited smaller invasive tumors and less lymph node involvement, than patients not taking the anxiolytic (Kleinerman et al., 1984). This retrospective study inspired our lab to explore the mechanism of benzodiazepine action in cancer cells. Benzodiazepine treatment in medulloblastoma and melanoma cells and respective murine models for these cancers impairs viability *via* mitochondrial depolarization and the intrinsic (mitochondrial) apoptotic pathway (Figure 4; Sengupta et al., 2014; Jonas et al., 2016; Kallay et al., 2019; Pomeranz Krummel et al., 2021). This anti-proliferative effect of a positive allosteric modulator, a benzodiazepine, is consistent with the earlier discussion of GABA_A_R and the maintenance of the stem cell niche. Given that benzodiazepines are often used as an anxiolytic during radiation treatment for a multitude of tumors, having this as a potential anti-cancer therapeutic in the “clinicians’ toolbox” would be valuable.

Importantly, benzodiazepines not only alone can impair the viability of cancer cells but also appear capable of sensitizing the cancer cells to radiation, chemotherapeutic, and an immune checkpoint inhibitor. If benzodiazepines can reduce the dose necessary to achieve the efficacy of these treatments, this may represent an approach to improve quality of life or reduce patient co-morbidities, because of these treatments. Underlying the basis for such sensitization by modulating the GABA_A_R may be due to an interaction of the receptor with an intracellular protein called GABARAP or GABA Type-A Receptor-Associated Protein (Pérez-Hernández et al., 2019). GABARAP has been reported to be an important contributor to the autophagosomes mediating autophagy, the process by which lysosomes degrade and recycle damaged organelles as a means for cells to control proliferation and the formation of radical inorganic species (Liu et al., 2016). This provides a putative mechanism linking the activation of GABA_A_R to the autophagy pathway.

Another potential pathway by which GABAergic signaling can influence tumor cell death is by modulating cells of the immune system. GABA_A_Rs appear to have roles in many immune cell subtypes, including T-cells, dendritic cells, macrophages, and neutrophils (Jin et al., 2013; Bhattacharya et al., 2021). Moreover, several immune cells have the capability of synthesizing and secreting GABA, including T-cells, macrophages, and dendritic cells (Bhat et al., 2010; Dionisio et al., 2011).

### Symptom Management

Treatment of cancer comes with a host of constitutional symptoms that significantly impact the quality of life of patients, including anxiety, insomnia, bone pain, and chemotherapy-induced nausea or vomiting (Triozzi et al., 1988). Anxiety may arise from the overall prospect of the disease itself or context-specific situations, such as radiotherapy. Benzodiazepines are commonly used as an anxiolytic during events such as radiation therapy. Bone pain can present as a consequence of a cancer metastasis. Nausea and vomiting are common side effects of chemotherapeutics. Regardless of the origin of symptoms, benzodiazepines and other GABA_A_R modulators are useful therapeutics, as they simultaneously treat all of the above symptoms.

Work in a rat model of CIBP found that GABA was downregulated in CIBP rats in combination with the upregulation of GABA transporter type-1 (GAT-1) in spinal cord astrocytes (Ge et al., 2019). Interestingly, administration of exogenous GAD and NO-711 (a GAT-1 specific-inhibitor) significantly reversed CIBP-induced mechanical allodynia (Ge et al., 2019). This suggests that the upregulation of GABAergic signaling has the potential to be an analgesic in CIBP. Moreover, given the effects that GABA agonism has been shown to have on tumor regression by Sengupta et al. (2014) this suggests a dual benefit to GABA treatment for cancer—addressing the symptoms and root cause simultaneously. This treatment must also be contextualized in light of the research by Neman et al. (2014) and Takehara et al. (2007) which implicated GABA signaling in tumor proliferation. Likely tissue-specific factors will determine the efficacy of GABA agonism or antagonism therapy.

## Conclusion

Members of the three ligand-gated ionotropic channel superfamilies are implicated in both the progression and symptomatology of a variety of tumors. Alterations in purinergic, glutamatergic, and Cys-loop signaling have been shown to increase tumor proliferation *via* induction of intracellular signaling pathways. Some preliminary research has been conducted in terms of both “antagonism” and “agonism” of these receptor subtypes and a resulting decrease in proliferation and/or migration of tumor cells both *in vitro* and *in vivo*. Aside from the effects on tumor proliferation, antagonism of these receptor subtypes can also be helpful in the management of cancer-associated symptoms, such as cancer-induced bone pain and seizures. Given the multifaceted nature of ligand-gated ionotropic receptors in cancer, modulating these systems may present a potent method to simultaneously improve symptoms and outcomes.

Most ion channel drug development up to this point has focused on small molecule and peptide modulators which have allosteric or direct modulation of the ligand-gated ion channel’s pore function. Initially, these compounds were found serendipitously, mostly from natural compounds (Kalia et al., 2015). However, as more structures of members of these receptor families were determined, biochemists were able to better rationally design compounds based on putative binding sites (Zhao et al., 2021). Recently, there is increased research into employing monoclonal antibodies against ligand-gated ionotropic channels. There is a Phase 1 clinical trial underway employing monoclonal antibodies against P2X_7_R to treat basal cell carcinoma (Biosceptre, 2016; Hutchings et al., 2018). Other clinical trials have focused on antagonizing the mGluR1 signaling pathway using riluzole with secondary outcomes being tumor shrinkage (Barbara Ann Karmanos Cancer Institute, 2013; Rutgers, The State University of New Jersey, 2014). However, these trials have been terminated due to a lack of funding and accrual. Clinical trials involving GABA_A_R remain sparse despite promising *in vitro* data.

There remains much to be discovered both in terms of mechanism and off-target effects of ligand-gated ionotropic channel synthetic modulators. In particular, either a positive or negative allosteric modulator in one cancer subtype may have a differing effect compared to the same compound in another cancer tissue. This may be a product of receptor subtype variants (i.e., splice variants) or differing intracellular signaling mechanisms. Regardless, targeting ligand-gated ionotropic channels remains a promising pathway to offering targeted therapeutics for cancer treatment.

## Author Contributions

RR and SS drafted and edited the manuscript. DB, DT, RC, DAPK, and SSG contributed to the writing and editing of the manuscript. All authors contributed to the article and approved the submitted version.

## Conflict of Interest

DAPK and SSG were co-founders of Amlal Phamaceuticals, Inc. DAPK is the President/CEO of Amlal Pharmaceuticals, Inc. The remaining authors declare that the research was conducted in the absence of any commercial or financial relationships that could be construed as a potential conflict of interest.

## Publisher’s Note

All claims expressed in this article are solely those of the authors and do not necessarily represent those of their affiliated organizations, or those of the publisher, the editors and the reviewers. Any product that may be evaluated in this article, or claim that may be made by its manufacturer, is not guaranteed or endorsed by the publisher.
